# *RICTOR* amplification is associated with Rictor membrane staining and does not correlate with PD-L1 expression in lung squamous cell carcinoma

**DOI:** 10.3389/pore.2024.1611593

**Published:** 2024-04-19

**Authors:** Ildikó Krencz, Dániel Sztankovics, Anna Sebestyén, Judit Pápay, Titanilla Dankó, Dorottya Moldvai, Elmar Lutz, Andras Khoor

**Affiliations:** ^1^ Department of Pathology and Experimental Cancer Research, Semmelweis University, Budapest, Hungary; ^2^ Department of Laboratory Medicine and Pathology, Mayo Clinic, Jacksonville, FL, United States

**Keywords:** *RICTOR* amplification, Rictor expression, mTORC2, PD-L1, lung squamous cell carcinoma

## Abstract

*RICTOR* gene, which encodes the scaffold protein of mTORC2, can be amplified in various tumor types, including squamous cell carcinoma (SCC) of the lung. *RICTOR* amplification can lead to hyperactivation of mTORC2 and may serve as a targetable genetic alteration, including in lung SCC patients with no PD-L1 expression who are not expected to benefit from immune checkpoint inhibitor therapy. This study aimed to compare *RICTOR* amplification detected by fluorescence *in situ* hybridization (FISH) with Rictor and PD-L1 protein expression detected by immunohistochemistry (IHC) in SCC of the lung. The study was complemented by analysis of the publicly available Lung Squamous Cell Carcinoma (TCGA, Firehose legacy) dataset. *RICTOR* amplification was observed in 20% of our cases and 16% of the lung SCC cases of the TCGA dataset. Rictor and PD-L1 expression was seen in 74% and 44% of the cases, respectively. Rictor IHC showed two staining patterns: membrane staining (16% of the cases) and cytoplasmic staining (58% of the cases). Rictor membrane staining predicted *RICTOR* amplification as detected by FISH with high specificity (95%) and sensitivity (70%). We did not find any correlation between *RICTOR* amplification and PD-L1 expression; *RICTOR* amplification was detected in 18% and 26% of PD-L1 positive and negative cases, respectively. The TCGA dataset analysis showed similar results; *RICTOR* copy number correlated with Rictor mRNA and protein expression but showed no association with PD-L1 mRNA and protein expression. In conclusion, the correlation between *RICTOR* amplification and Rictor membrane staining suggests that the latter can potentially be used as a surrogate marker to identify lung SCC cases with *RICTOR* amplification. Since a significant proportion of PD-L1 negative SCC cases harbor *RICTOR* amplification, analyzing PD-L1 negative tumors by *RICTOR* FISH or Rictor IHC can help select patients who may benefit from mTORC2 inhibitor therapy.

## Introduction

Despite the significant advances that have been made in the diagnosis and treatment of non-small cell lung cancer (NSCLC) in the past decades, it remains the leading cause of cancer death worldwide [1]. Among NSCLCs, squamous cell carcinoma (SCC) is one of the most common histological subtypes, accounting for 30%–35% of all NSCLC cases [2].

Lung SCC is strongly associated with tobacco smoking and arises mostly in the central part of the lungs [3]. There is growing evidence that it is more heterogeneous than it is suggested by its three histologic subtypes (keratinizing, non-keratinizing, and basaloid) [4].

While there have been significant breakthroughs in targeted therapies for lung adenocarcinoma, most patients with advanced SCC are treated with immune checkpoint inhibitors (ICIs), platinum-based chemotherapy, or a combination of the two [5]. For SCCs with no PD-L1 expression, platinum-based chemotherapy remains the main treatment option [6]. Therefore, there is an urgent need to develop new targeted therapies for patients with SCC.

**FIGURE 1:**
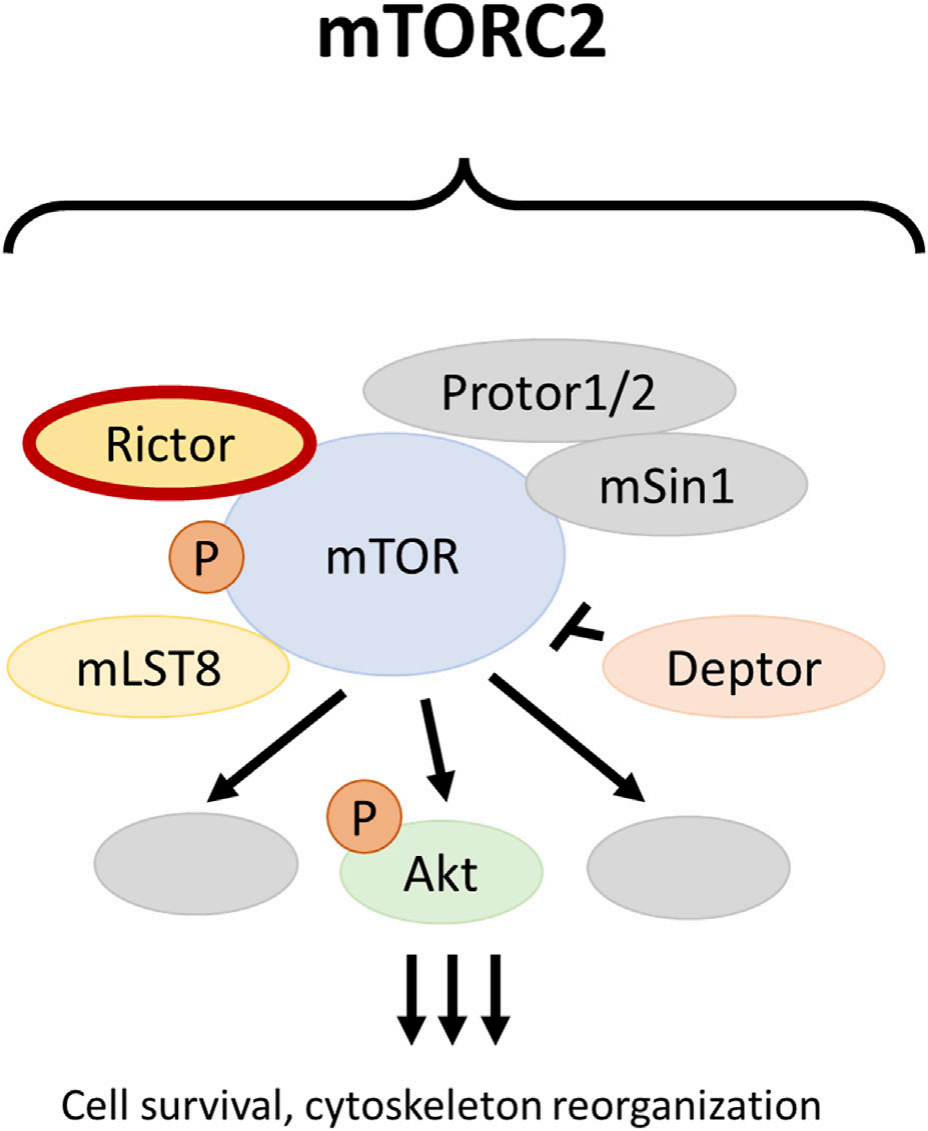
The mTOR complex 2. The *RICTOR* gene encodes for the scaffold protein of mTORC2 and can be amplified in different malignancies, including squamous cell carcinoma of the lung.

Protein complexes mTOR complex 1 (mTORC1) and mTOR complex 2 (mTORC2) are integral components of the phosphatidylinositol 3-kinase (PI3K)/protein kinase B (Akt)/mammalian target of rapamycin (mTOR) pathway. The main functions of mTORC1 include the regulation of cell growth and proliferation, whereas mTORC2 plays essential roles in cytoskeletal reorganization and cell survival [7]. Rictor (encoded by the *RICTOR* gene) is the scaffold protein of mTORC2 (Figure 1). Amplification of *RICTOR* has been reported in various cancer types, including approximately 10% of lung SCCs [8–10]. Like *PIK3CA* copy number gain and *PTEN* loss, *RICTOR* amplification affects the PI3K/Akt/mTOR pathway. *PIK3CA* copy number gain and *PTEN* loss mostly alter mTORC1 activity, whereas *RICTOR* amplification primarily results in the alteration of mTORC2 [11].

*RICTOR* fluorescence *in situ* hybridization (FISH) is considered the diagnostic standard for the detection of *RICTOR* amplification [8]. Immunohistochemistry (IHC) is an acceptable, cost-effective alternative to detect certain molecular alterations, including *ALK* and *ROS1* rearrangements [12, 13]. However, Rictor IHC has never been compared to *RICTOR* FISH in the SCC of the lung.

The purpose of this study was to compare *RICTOR* amplification as detected by FISH with Rictor expression as detected by IHC in SCC of the lung. Since PD-L1 negative tumors are typically ineligible for immune checkpoint inhibitor therapy, finding a potentially targetable genetic alteration such as *RICTOR* amplification is especially important for patients with PD-L1 negative advanced SCC. Therefore, we also examined the relationship between *RICTOR* amplification and PD-L1 expression in these tumors.

## Materials and methods

### Patients

A total of 50 samples, including 40 surgical resection specimens and 10 small biopsies of lung SCC cases diagnosed between 1 January 2002 and 31 July 2020 were analyzed in our study. Only primary tumors with SCC histology were included. The tumors were re-reviewed and reclassified according to the 2021 World Health Organization Classification of Thoracic Tumors [14]. None of the patients received neoadjuvant therapy before surgery or biopsy. Clinicopathological data are summarized in Table 1. The study was approved by the Mayo Clinic Institutional Review Board (ID: 18-001887) and the Institutional Ethical Review Board of Semmelweis University (SE KREB 216/2020).

**TABLE 1:** Clinicopathological data.

Characteristics		N (%)
Total no. of the cases		50 (100%)
Age	<65 years	12 (24%)
	≥65 years	38 (76%)
Sex	Male	30 (60%)
	Female	20 (40%)
Histological subtype	Non-keratinizing	31 (62%)
	Keratinizing	16 (32%)
	Basaloid	3 (6%)
Grade	Grade 1	1 (2%)
	Grade 2	33 (66%)
	Grade 3	16 (32%)
Stage	IA-B	25 (50%)
	IIA-B	10 (20%)
	IIIA-B	9 (18%)
	N.A.	6 (12%)
Procedure	Surgical resection	40 (80%)
	Small biopsy	10 (20%)

Abbreviations: N.A., not available.

### Fluorescence *in situ* hybridization reaction

FISH was performed on 4–µm–thick sections of the formalin-fixed paraffin-embedded tissue blocks. After pretreatment with Vysis IntelliFISH Pretreatment SSC Solution (Abbott Molecular, Des Plaines, IL, United States) at 79°C for 25 min, deparaffinized sections were digested using Vysis IntelliFISH Protease (Abbott Molecular) at 38°C for 25 min. Sections were then exposed to a mixture of *RICTOR* (#RICTOR-20-OR; Empire Genomics, Buffalo, NY, United States, labeled in orange) and chromosome 5 (Chr5) control (#CHR05-10-GR; Empire Genomics, labeled in green) probes and Vysis IntelliFISH Hybridization Buffer (Abbott Molecular) at 37°C for 2 h. Posthybridization wash was performed at 73°C for 3 min (Vysis IntelliFISH Post-Hybridization Buffer; Abbott Molecular). After air drying, sections were counterstained with DAPI I (Abbott Molecular).

### Fluorescence *in situ* hybridization analysis

**FIGURE 2:**
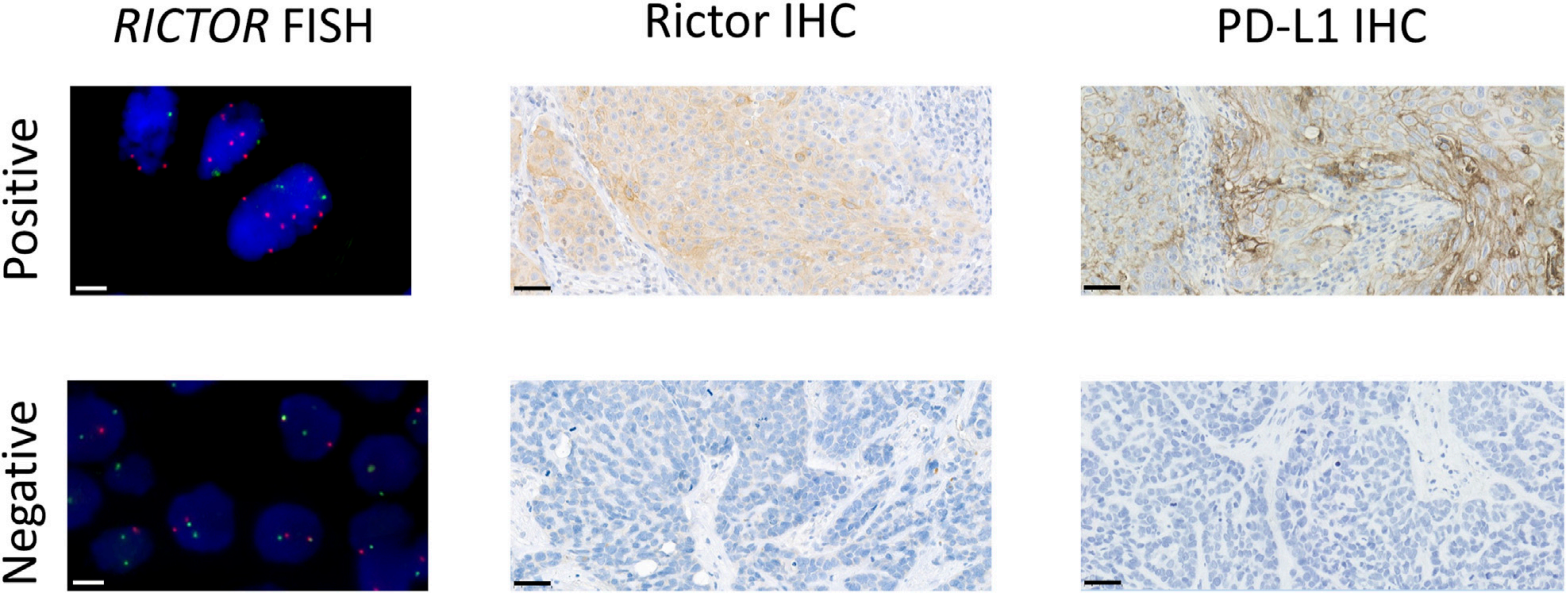
Representative images of *RICTOR* FISH, Rictor IHC, and PD-L1 IHC in lung SCC cases. Representative images of *RICTOR* FISH (*RICTOR* and *Chr5* are labeled in orange and green, respectively), Rictor IHC, and PD-L1 IHC in lung SCC cases. The scale bar indicates 10 μm on FISH and 50 μm on IHC images.

Slides were examined under a fluorescence microscope (DM5500 B; Leica, Wetzlar, Germany) focusing on hot spot areas. For each tumor, 30 tumor cell nuclei were evaluated in at least 2 different areas by 2 technicians. Both the mean number of signals per nucleus and the *RICTOR*/Chr5 control ratio were determined. A *RICTOR* copy number of 4 or more and a *RICTOR*/Chr5 ratio of 2 or more were considered positive for *RICTOR* amplification. Otherwise, the case was considered negative (see Figure 2). Images were captured using CytoVision software (Leica).

### Immunohistochemical staining

Rictor IHC was performed on 4–µm–thick sections of the same blocks. After deparaffinization and blocking endogenous peroxidases, antigen retrieval was performed for 30 min (10 mM citrate, pH 6.0) in a pressure cooker. Slides were then incubated with an anti-Rictor (1:1000; #A500-002A; Bethyl Laboratories, Montgomery, TX, United States) primary antibody. Vectastain Universal Elite ABC HRP Kit (Vector Laboratories, Newark, CA, United States) secondary detection system and DAB (Agilent, Santa Clara, CA, United States) chromogen were used to visualize the reactions. The sections were counterstained with hematoxylin.

PD-L1 IHC 22C3 PharmDx reactions were performed on an automated immunostainer (Leica BOND-III; Leica) using the EnVision FLEX visualization system (Agilent) according to the manufacturer’s protocols.

### Evaluation of immunohistochemistry

PD-L1 membrane expression was assessed using Tumor Proportion Score (TPS) and was classified into TPS < 1%, TPS 1%–49%, and TPS ≥ 50% categories [15, 16]. A TPS ≥ 1% was considered positive (see Figure 2).

Since there had been no standardized way of analyzing Rictor expression, we evaluated both membrane and cytoplasmic stainings. In the presence of any membrane positivity, the pattern was considered membrane staining. The percentage of stained tumor cells was categorized similarly to that of PD-L1, using TPS < 1%, TPS 1%–49%, and TPS ≥ 50% categories.

### Data collection from the cancer genome atlas

Correlations among copy number alterations for *RICTOR* as well as mRNA (mRNA Seq V2 RSEM) and protein (reverse-phase protein array, RPPA) expression of Rictor and PD-L1 (CD274) was analyzed in the Lung Squamous Cell Carcinoma (TCGA, Firehose Legacy) dataset [17, 18]. The dataset contains the data of 511 lung SCC patients and was accessed through cBioPortal (accessed on 30th March 2023^1^). The association between *RICTOR* amplification and clinicopathological data was also studied.

### Statistical analysis

Statistical analysis was performed using IBM SPSS Statistics software (version 22; SPSS Inc., Chicago, IL, United States). Descriptive data are expressed as numbers (percentages). Associations with the clinicopathological data were analyzed by the chi-square test. Correlations were evaluated using Spearman correlation. The Kaplan-Meier method was used for the survival analysis. The mean follow-up time was 1,091 days. Survival curves were compared by the log-rank method. A *p ≤* 0.05 was defined as statistically significant.

## Results

### *RICTOR* amplification in lung SCC

*RICTOR* amplification was observed in 10 of 50 cases (20%). The mean (range) *RICTOR* copy number per tumor cells was 6.26 (4.48–9.69) in the amplified cases and 2.90 (1.62–4.20) in the non-amplified cases. The mean (range) *Chr5* copy number per tumor cells was 1.97 (1.27–2.89) in the amplified cases and 1.64 (1.10–2.52) in the non-amplified cases.

### Immunohistochemical expression of Rictor in lung SCC

**FIGURE 3:**
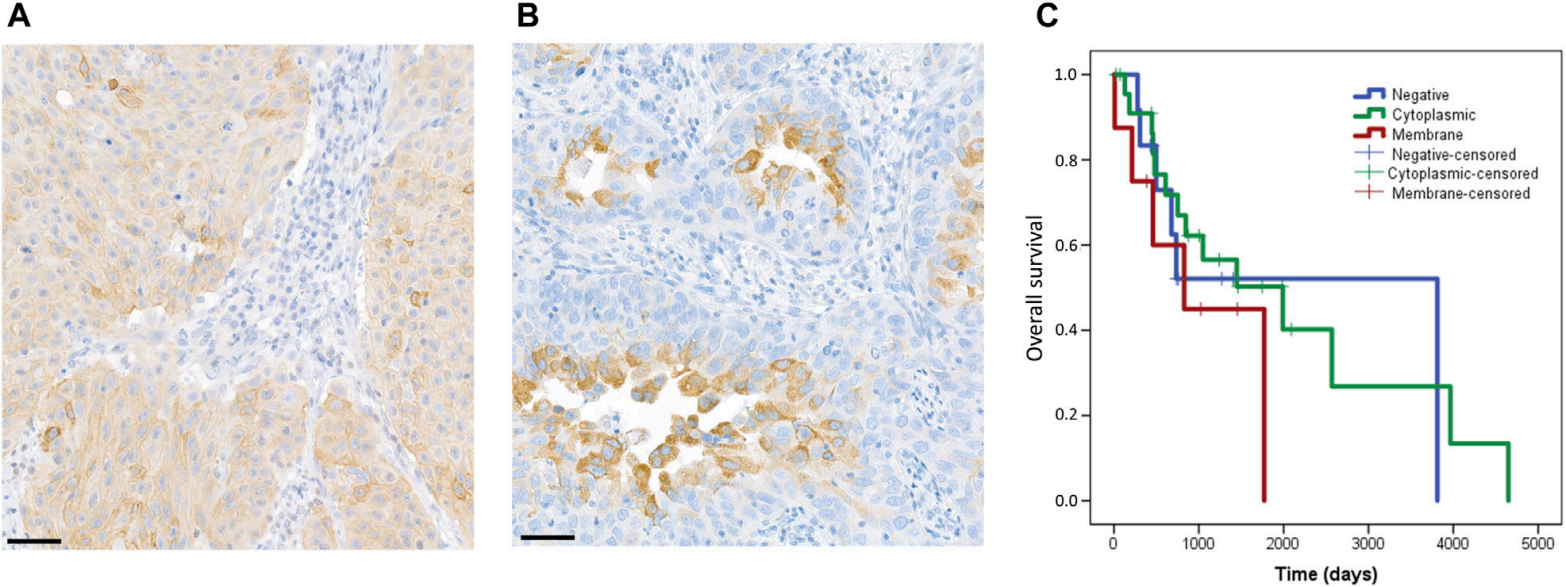
Staining pattern of the Rictor protein and correlation with patient’s overall survival. In our study, we evaluated membrane and cytoplasmic staining separately. The figure shows representative images for membrane **(A)** and cytoplasmic **(B)** staining. The scale bar indicates 50 μm. Correlation of Rictor staining pattern with overall survival of patients **(C)**. Rictor membrane staining (red) was associated with slightly shorter overall survival as compared to cytoplasmic staining (green) and negative cases (blue).

Assessment of Rictor staining had not been standardized previously. Therefore, we separately analyzed membrane staining (Figure 3A) and cytoplasmic staining (Figure 3B) in our study. Membrane staining was observed in 8 cases (16%) and cytoplasmic staining in 29 cases (58%) (Table 2). No Rictor expression was seen in 13 cases (26%).

**TABLE 2:** Immunohistochemical expression of Rictor in squamous cell carcinoma.

Rictor expression	TPS (%)	N (%)
Negative	<1	13 (26)
Positive membrane	1–49	3 (6)
	≥50	5 (10)
Positive cytoplasmic	1–49	27 (54)
	≥50	2 (4)
Total		50 (100)

### Immunohistochemical expression of PD-L1 in lung SCC

IHC for PD-L1 was positive in 22 of 50 cases (44%) (Table 3). TPS was 1%–49% in 17 cases and was ≥50% in 5 cases. The remaining 28 cases (56%) were negative.

**TABLE 3:** Immunohistochemical expression of PD-L1 in squamous cell carcinoma.

PD-L1 expression	TPS (%)	N (%)
Negative	<1	28 (56)
Positive	1–49	17 (34)
	≥50	5 (10)
Total		46 (100)

### Concordance between *RICTOR* FISH and Rictor expression

The sensitivity, specificity, and negative and positive predictive values of various Rictor staining patterns are shown in Table 4. Rictor membrane staining showed the highest specificity for *RICTOR* amplification (95%). The negative predictive value of Rictor membrane staining was also high (93%).

**TABLE 4:** Concordance of *RICTOR* FISH and Rictor immunohistochemical results.

	Rictor membrane staining (%)	Rictor cytoplasmic staining (%)	Any Rictor staining (%)
Sensitivity	70	20	90
Specificity	95	35	30
Negative predictive value	93	64	92
Positive predictive value	78	7	24

### Concordance between *RICTOR* FISH/Rictor expression and PD-L1 expression

*RICTOR* amplification was detected in 4 (18%) PD-L1 positive cases and 5 (26%) PD-L1 negative cases, and there was no correlation between *RICTOR* amplification and PD-L1 expression. Similarly, we found no significant correlation between Rictor and PD-L1 expression.

### Correlation of *RICTOR* amplification, Rictor and PD-L1 expression with clinicopathological data and survival

Neither FISH, nor immunohistochemical results showed a correlation with clinicopathological data (including age, sex, histological subtype, grade, and stage). Patients with Rictor membrane staining had a slightly shorter overall survival as compared to patients with Rictor cytoplasmic staining (*p =* 0.229) or no Rictor staining (*p =* 0.505) (Figure 3C). However, these results were not statistically significant. *RICTOR* amplification and PD-L1 expression did not correlate with survival.

### Associations between *RICTOR* amplification, Rictor, and PD-L1 mRNA and protein expression in the lung squamous cell carcinoma (TCGA, Firehose Legacy) dataset

**FIGURE 4:**
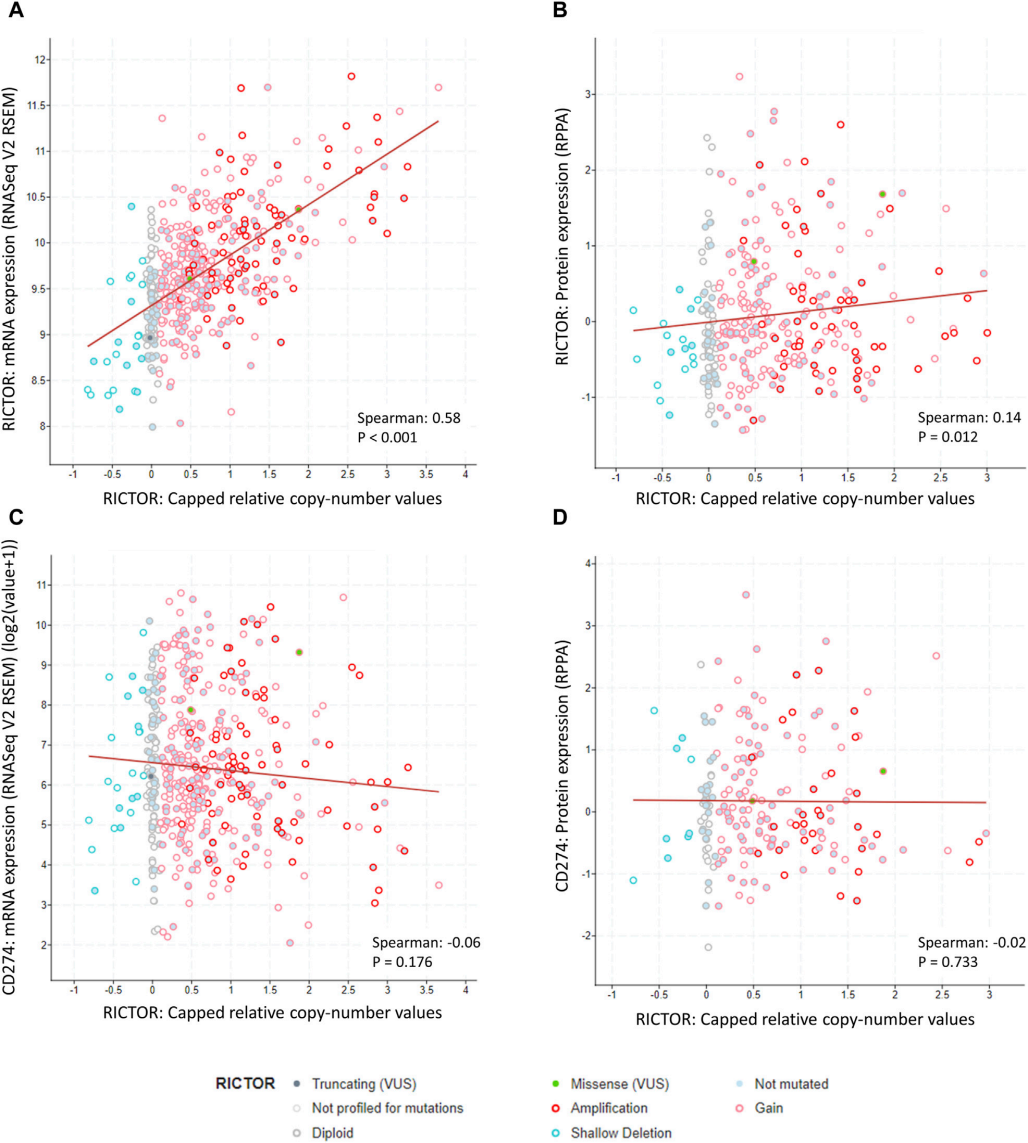
Correlation between *RICTOR* copy number, *RICTOR* and *CD274* (PD-L1) mRNA, and Rictor and CD274 (PD-L1) protein expression in the Lung Squamous Cell Carcinoma (TCGA, Firehose Legacy) dataset. **(A)** Strong positive correlation between *RICTOR* copy number and *RICTOR* mRNA expression. **(B)** Weak positive correlation between *RICTOR* copy number and Rictor protein expression. **(C)** No correlation between *RICTOR* copy number and *CD274* mRNA and **(D)** PD-L1 protein expression. In addition, to copy number variations, missense (2 patients, *RICTOR* R1299G, *RICTOR* E1633Q) and truncating (1 patient, *RICTOR* Q569*) mutations were also present in the *RICTOR* gene, without any known significance.

To strengthen our results, we also performed a similar analysis using data from the Lung Squamous Cell Carcinoma (TCGA, Firehose Legacy) dataset (*N* = 511). 501 of these samples were profiled for *RICTOR* copy number alterations, and *RICTOR* amplification was seen in 16% of the cases. We observed a strong positive correlation between *RICTOR* copy number and *RICTOR* mRNA expression (Spearman’s R = 0.58, *p <* 0.001; 498 samples with data in both profiles, see Figure 4A). A weaker positive correlation was detected between *RICTOR* copy number and Rictor protein expression (Spearman’s R = 0.14, *p =* 0.012; 326 samples with data in both profiles, see Figure 4B). However, there was no available information about the intracellular localization of Rictor protein expression. In correlation with our FISH and immunohistochemical analysis, we found no association between *RICTOR* copy number and PD-L1 (*CD274*) mRNA expression (Spearman’s R= −0.06, *p =* 0.176; 498 samples with data in both profiles, see Figure 4C) or PD-L1 protein expression (Spearman’s R= −0.02, *p =* 0.733; 194 samples with data in both profiles, see Figure 4D).

### Associations between *RICTOR* amplification and clinicopathological data in the lung squamous cell carcinoma (TCGA, Firehose Legacy) dataset

The TCGA data analysis confirmed that there is no association between *RICTOR* amplification and overall survival in lung SCC patients (*p =* 0.201). Despite most of the cases have been categorized as Squamous Cell Carcinoma NOS based on histology (ICD-O-3: 8070/3), *RICTOR* amplification was seen more frequently in basaloid SCCs (6.33% of the *RICTOR*-amplified cases and 2.13% of the non-amplified cases had basaloid histology). In contrast, *RICTOR* amplification was absent in keratinizing SCCs (none of the *RICTOR*-amplified cases, but 3.08% of the non-amplified cases were keratinizing SCCs). Additionally, we could not find any association between *RICTOR* amplification and any other genetic drivers (*ALK* translocation and *KRAS* mutation status were available in 286 and 15 patients, respectively) or tumor mutational burden.

## Discussion

This is the first study to compare *RICTOR* amplification and Rictor expression in human lung SCC. Using FISH, we observed *RICTOR* amplification in 20% of the cases. This is slightly higher than *RICTOR* amplification in the Lung Squamous Cell Carcinoma (TGCA, Firehose Legacy) dataset or in previous database analyses, where it has been found in 16% and 7.4%–15% of SCC cases, respectively [19, 20]. The difference among various studies may be due to differences in study populations and methods used to detect *RICTOR* amplification. An additional explanation for these differences is the high heterogeneity of RICTOR amplification and overexpression, which has been described previously [21] and was also found in our study. Given the potential intratumoral heterogeneity of both Rictor and PD-L1 staining [21–23], we performed both FISH and IHC analysis on whole slides of the tissue blocks, which allows for a higher probability of detecting areas of *RICTOR* amplification and overexpression of a heterogeneous tumor.

Rictor is the scaffold protein for mTORC2, a protein complex interconnected with the PI3K/Akt/mTOR pathway [24]. The PI3K/Akt/mTOR pathway regulates several essential cellular functions related to environmental signals, including cell proliferation, growth, homeostasis, and metabolism.

Subcellular localization of the PI3K/Akt/mTOR pathway components has been evaluated in various studies. As the first step in the pathway, PI3K generates phosphatidylinositol-3,4,5-triphosphate (PIP3), which binds the pleckstrin homology domain of Akt, recruiting it to the plasma membrane. Once Akt is localized to the plasma membrane, mTORC2 can phosphorylate its Ser473 site, thereby facilitating activation of the PI3K/Akt/mTOR pathway [20, 25, 26]. In contrast, some studies have demonstrated that mTORC2 can also phosphorylate its substrates (e.g., serum/glucocorticoid regulated kinase 1) using intracellular membranes and subcellular spaces such as the endoplasmic reticulum and the perinuclear compartment [25, 26]. Additionally, it is also known that Rictor can interact with integrin-linked kinase (ILK), a protein with a multifunctional role in cell-matrix interactions and that the Rictor-ILK complex may contribute to the phosphorylation of Akt on Ser473 [27, 28]. Consistent with these data, a recent study has described more than 200 proteins that can interact with mTORC2 and possibly regulate its activity and localization [29]. We observed both membrane and cytoplasmic staining for Rictor in our study. Interestingly, in *RICTOR* amplified cases, Rictor was localized primarily to the plasma membrane, whereas, in non-amplified cases, we found predominantly cytoplasmic staining consistent with intracellular localization.

Previous studies have revealed a positive correlation between *RICTOR* mRNA expression and DNA copy number in NSCLC [20], which was confirmed with our database analysis. Similarly, we observed a positive correlation between Rictor protein expression and *RICTOR* gene amplification. However, this correlation was strongly dependent on subcellular localization. Rictor membrane staining showed high specificity (95%) and moderate to high sensitivity (77%) for *RICTOR* amplification, whereas the specificity and sensitivity of Rictor cytoplasmic staining were much lower (38% and 11%, respectively). Therefore, our recommendation is to use Rictor membrane staining as a surrogate marker for *RICTOR* gene amplification.

Despite breakthroughs in targeted therapies for lung adenocarcinoma in the past decades, limited progress has been made in the treatment of lung SCC. This seems to be due to the lower frequency of targetable genetic alterations and the less favorable response to small-molecule tyrosine kinase inhibitors [30, 31]. The PI3K/Akt/mTOR pathway represents a potential therapeutic target [32]. The activity of mTORC2 can be inhibited by dual mTOR kinase inhibitors, which affect both mTORC1 and mTORC2 (e.g., vistusertib and sapanisertib). They have been used to treat various types of lung cancer, including SCC of the lung, in early-phase clinical trials [9, 11]. In most of these studies, patients have not been stratified based on the presence of *RICTOR* amplification. A selective mTORC2 inhibitor (JR-AB2-011), which blocks the interaction between Rictor and mTOR, is already available and has been used with promising results in preclinical trials [33, 34]. Selecting patients with *RICTOR*-amplified tumors may improve the results.

**FIGURE 5:**
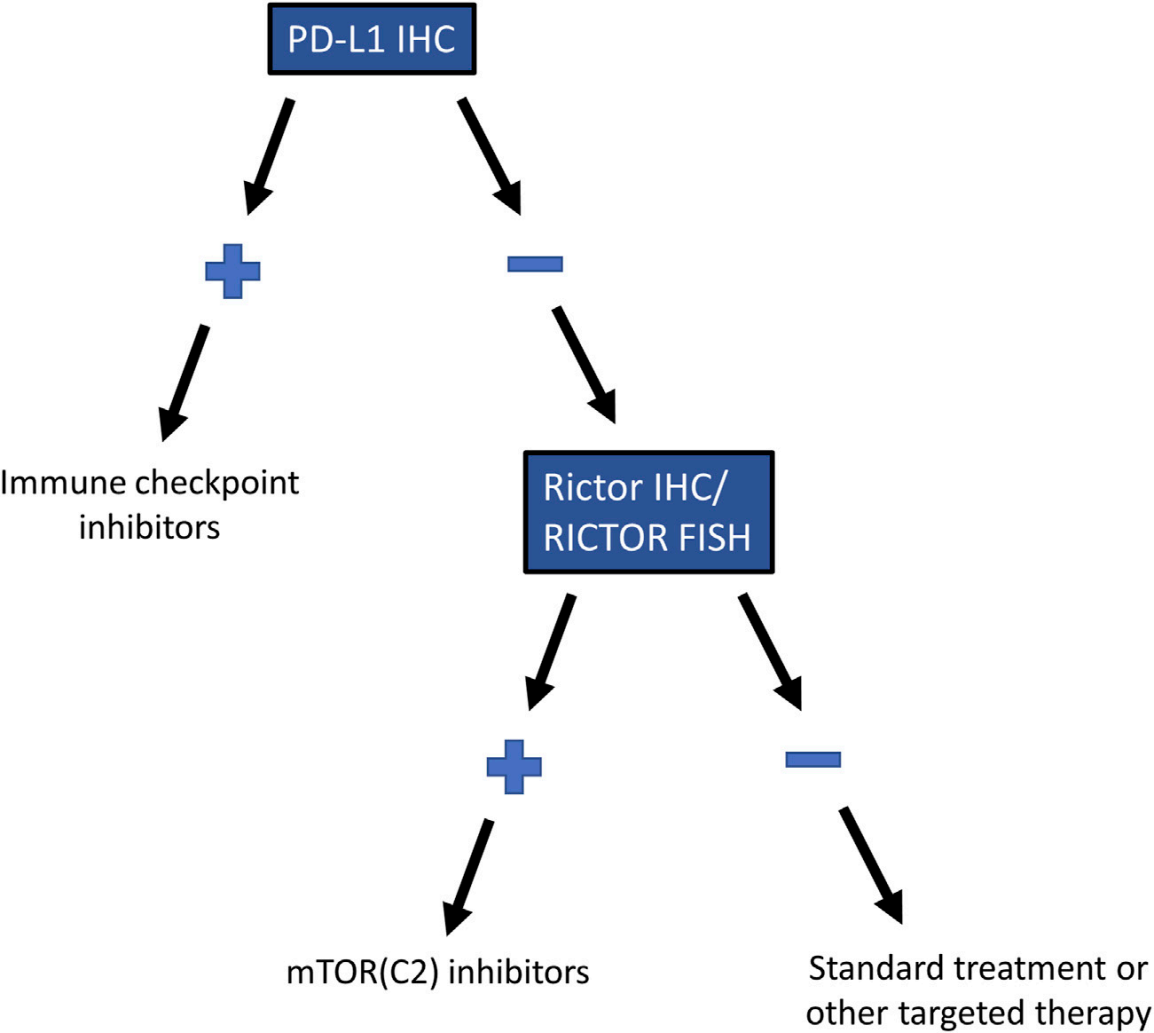
Recommended testing algorithm for *RICTOR* amplification in lung SCC. Testing algorithm for selecting patients with *RICTOR* amplification who are not eligible for immune checkpoint inhibitor treatment but may benefit from mTOR (C2) inhibitors.

In advanced cases of SCC of the lung, ICIs represent the only widely used therapeutic option besides radiochemotherapy [6]. Similar to a previous study, which has found no association between gene alterations and PD-L1 expression in advanced lung SCC [35], we observed no association between PD-L1 expression, a predictive marker for the efficacy of ICIs, and *RICTOR* amplification or Rictor expression. *RICTOR* amplification occurred in PD-L1 positive and negative cases with similar frequency, suggesting that a significant number of the PD-L1 negative cases can be eligible for mTORC2 inhibitor therapy (Figure 5). Additionally, *RICTOR* amplification has been speculated to be responsible for the resistance against chemotherapy, tyrosine kinase inhibitors, and ICIs, leading to tumor progression in various types of cancer [36–38]. Therefore, analyzing *RICTOR* amplification in lung SCC patients who no longer respond to ICIs can reveal a new targetable alteration in this subpopulation of patients.

Several studies have shown a correlation between mTORC2 activity and metastatic disease [33, 39, 40]. This can result in decreased survival in *RICTOR-*amplified cases [41]. However, similar to our previous observation in small cell lung carcinoma [8], we did not detect a significant correlation between *RICTOR* amplification and unfavorable clinical outcomes in SCC of the lung. Nevertheless, Rictor membrane staining, which might be directly associated with Akt phosphorylation and thereby mTORC2 activity, did show slightly lower overall survival. These results might be clarified in a study with a larger sample size focused on this particular question.

In conclusion, our findings indicate that a significant proportion of lung SCCs, including PD-L1-negative cases, harbor *RICTOR* amplification. Despite the initial failures in early-phase clinical trials, one of the key messages of our study is that stratifying patients based on the presence of *RICTOR* amplification or Rictor overexpression may improve the results. With the advent of next-generation inhibitors that can selectively target mTORC2, the importance of this topic is emerging. Rictor membrane staining can predict *RICTOR* amplification as detected by FISH with high specificity and sensitivity; therefore, it can potentially be used as a surrogate marker to identify *RICTOR*-amplified lung SCCs. Analysis of lung SCCs by *RICTOR* FISH or Rictor IHC, especially in PD-L1-negative cases, can help select patients who may benefit from mTORC2 inhibitor therapy.

## Data Availability

The raw data supporting the conclusion of this article will be made available by the authors, without undue reservation.

## Footnotes

1
www.cbioportal.org
